# Hippocampal Development and Epilepsy: Insights from Organoid Models

**DOI:** 10.3390/brainsci15111231

**Published:** 2025-11-16

**Authors:** Jin Joo, Woo Sub Yang, Hyun Jung Koh

**Affiliations:** 1 Department of Anesthesiology and Pain Medicine, Seoul St. Mary’s Hospital, College of Medicine, The Catholic University of Korea, Seoul 06591, Republic of Korea; jiyo1004@catholic.ac.kr; 2 Department of Genetics, Yale Stem Cell Center, Yale School of Medicine, New Haven, CT 06520, USA; woosubyang@gmail.com

**Keywords:** brain organoid, cognitive function, developing hippocampus, epilepsy, hippocampal organoid

## Abstract

The hippocampus is a crucial component of the human brain. It is located on the medial side of the temporal lobe and is connected to the limbic system, influencing memory and cognitive function. The critical functions of the hippocampus have a profound impact on an individual’s overall ability to maintain daily life functioning. In adults, hippocampal damage impairs cognitive functions, including memory, learning, and emotional regulation. It is associated with conditions such as memory impairment, Alzheimer’s disease, various forms of dementia, depression, and stress-related disorders. Damage to the developing hippocampus can have broad and profound, leading to deficits in memory development, language acquisition, and behavioral and emotional regulation, thereby impairing the individual’s ability to maintain normal daily functioning. One of the major factors affecting hippocampal development is epilepsy. Therefore, identifying the mechanism underlying epilepsy-induced hippocampal damage and developing therapeutic strategies to reduce or prevent epileptic events that significantly impair hippocampal maturation are of critical importance. Numerous studies have been conducted in this regard, and given the challenges of directly studying the human brain, organoid-based research approaches have gained increasing attention and widespread application. In particular, hippocampal organoids have emerged as valuable models for investigating various hippocampal functions; however, definitive findings have yet to be established. Therefore, elucidating the structural characteristics and underlying mechanisms of epilepsy using hippocampal organoids, and exploring potential strategies to mitigate its effects remains an important direction for future research.

## 1. Introduction

The hippocampus is a crucial brain structure primarily responsible for forming, organizing, and consolidating new memories and supporting learning and spatial navigation. It converts short-term experiences into long-term memories and plays a central role in recalling facts, events, and spatial environments [1]. Proper hippocampal function is fundamental for maintaining normal cognition and behavior throughout life.

Because hippocampal development continues after birth, early-life damage can interfere with its structural and functional maturation. Such developmental disturbances may increase vulnerability to various neurological disorders later in life, including Alzheimer’s disease, Huntington’s disease, Parkinson’s disease, multiple sclerosis, and frontotemporal dementia [2,3].

Among these conditions, epilepsy poses a significant threat to hippocampal development, especially during early stages of life. Seizures during critical developmental periods can disrupt normal neurogenesis, alter synaptic connectivity, and lead to persistent deficits in memory, cognition, and behavior [4,5,6]. While epilepsy in adulthood can also contribute to neurodegenerative progression, its impact is far more pronounced when it occurs during early brain development, when neuronal circuits are still maturing.

Current epilepsy management primarily focuses on controlling or reducing seizures and improving patients’ quality of life through pharmacological therapy, surgical interventions, dietary approaches, and neuromodulation techniques in drug-resistant cases. However, despite these advances, it remains unclear whether current treatments can prevent or reverse the structural and functional damage caused by epilepsy.

Direct investigation of the human brain poses considerable challenges, and most existing knowledge has been derived from animal models, which cannot fully replicate human neurodevelopmental processes. Recent advances in stem cell technology have enabled the generation of three-dimensional human brain organoids that closely mimic the structural and functional architecture of the developing brain. These organoids provide a promising platform for studying disease mechanisms that are otherwise inaccessible in vivo.

This review aims to summarize the fundamental structure and developmental processes of the hippocampus and to explore its associations with epilepsy and related neurological disorders. Furthermore, we highlight recent progress in hippocampal organoid research and its potential applications in elucidating epileptogenesis and developing future therapeutic strategies.

## 2. Topical Sections

### 2.1. Search Strategy and Selection Criteria

The literature search was conducted using the PubMed, Embase, Scopus, and Google Scholar databases, covering studies published from 2000 onwards. The following search terms were used: “hippocampus” *AND* “anatomy and function” *AND* “developmental hippocampus” *AND* “epilepsy” *AND/OR* “brain organoid” *AND* “hippocampal organoid”. Although this was not a systematic review, references were selected based on their relevance to hippocampal structure, development, and epilepsy related mechanisms.

### 2.2. Structure, Development, and Functional Dynamics of the Hippocampus

The hippocampus is a paired, seahorse-shaped structure located deep within the medial temporal lobe of each cerebral hemisphere. As a key component of the limbic system, it comprises three major subregions; the hippocampus proper (Ammon’s horn or Cornu Ammonis; CA1–CA4), the dentate gyrus (DG), and the subiculum. Although CA4 was initially considered a distinct field, it is now generally considered part of the dentate gyrus. Among these subfields, CA1 and subiculum form extensive reciprocal connections with the entorhinal cortex, which serves as the principal interface between the hippocampus and the neocortex [7,8]. Major afferent inputs originate from the entorhinal cortex, septal area, prefrontal cortex, and anterior cingulate gyrus, whereas efferent projections target the septal nuclei, thalamus, hypothalamus, and cingulate cortex [7].

Functionally, the hippocampus plays a central role in converting short-term experiences into long-term memories, particularly in the formation of declarative and spatial memories related to facts, events, and navigation [9]. It supports memory consolidation during sleep and contributes to the formation of cognitive maps, which are essential for spatial orientation and learning.

The hippocampus begins to develop early in gestation, with distinct subregional architecture evident by 8–9 weeks of gestation. Folding and inversion of its key subregions, the DG and CA fields, occur between 13 and 16 weeks, and by 18–20 weeks, the hippocampus resembles its adult form. Structural maturation continues throughout the third trimester and into infancy [10]. From birth to two years of age, hippocampal volume more than doubles, followed by slower growth and pruning, particularly within the DG, during early childhood [11]. Maturation proceeds into adolescence, supported by progressive myelination and dendritic elaboration, although developmental timing differs among subfields [12].

Each hippocampal subregion follows a distinct developmental trajectory and assumes specialized functions. The DG is a major site of granule cell proliferation and the most active region of neurogenesis during development. It receives input from the entorhinal cortex, and continuous neurogenesis and circuit formation from early childhood into adulthood support new memory formation and pattern separation [13]. The CA regions, predominantly comprising pyramidal neurons, form the principal excitatory circuitry of the hippocampus [14]. The CA1 subfield functions as the primary output region, integrating processed information and relaying it to neocortical and subcortical targets; completion of dendritic branching and myelination in CA1 coincides with the emergence of complex spatial and episodic memory functions [13]. The CA3 subfield, essential for pattern completion, enables retrieval of stored memories from partial cues, with this capacity maturing in parallel with the establishment of recurrent excitatory circuits [15]. The subiculum acts as an integrative hub, channeling hippocampal output to cortical and limbic regions, and undergoes increased myelination during late adolescence, reinforcing its role in memory consolidation and cognitive flexibility [13].

Different hippocampal subfields exhibit distinct structural and functional trajectories across the lifespan. The CA1 and CA2 regions acquire mature functional roles gradually from childhood into early adulthood, followed by a linear decline in volume throughout aging. In contrast, the CA3 and DG subfields display nonlinear developmental patterns with prolonged maturation and later stabilization, particularly relevant to spatial memory and pattern separation functions [16]. The CA1 region is strongly associated with verbal and episodic memory processing, and continued dendritic remodeling and myelination support its role in higher cognitive function and complex memory retrieval [17]. The CA3 subfield, responsible for associative memory and pattern completion, continues to strengthen during adolescence and adulthood [15]. These region-specific trajectories contribute to the selective vulnerability of hippocampal circuits in age-related cognitive decline and neurodegenerative disorders.

### 2.3. Pathophysiological Changes in the Hippocampus in Epilepsy

Epilepsy frequently arises within a localized cortical region in which neuronal networks become hyperexcitable and prone to excessive synchronized firing, a phenomenon termed *ictogenesis*. Increasingly, epilepsy is recognized as a disorder of distributed brain networks rather than a focal disease. This broader view explains the cognitive and structural alterations often observed in regions distant from the primary seizure focus [18]. The medial temporal lobe structures—including the entorhinal cortex, amygdala, and para-hippocampal gyrus— closely interact with the hippocampus, collectively contributing to seizure initiation and propagation [19,20].

The underlying causes of epilepsy are often multifactorial or remain unidentified [8]. Seizures have been shown to induce aberrant neurogenesis within the hippocampus, leading to the formation of dysfunctional neuronal circuits that compromise normal hippocampal function [21]. Owing to its distinct cytoarchitecture and high intrinsic excitability, the hippocampus—particularly the CA1 and CA3 regions—is highly susceptible to seizure generation and is regarded as a primary locus of temporal lobe epilepsy (TLE) [20].

High-frequency oscillations (HFOs) within the hippocampus are established biomarkers of epileptogenic networks. These oscillations precede seizure onset and reflect abnormal synchronization among excitatory neuronal populations [20]. Seizures originating in the hippocampus trigger a cascade of neurophysiologic alterations that culminate in chronic epilepsy (*epileptogenesis*), characterized by spontaneous, recurrent seizures and persistent circuit remodeling [22]. Repeated hippocampal seizures promote widespread reorganization of cortical–subcortical connectivity, reinforcing seizure propagation pathways and sustaining the chronic epileptic state [22].

Hippocampal sclerosis—a common pathological hallmark of TLE—is strongly associated with recurrent seizure activity [19]. It is characterized by neuronal loss, gliosis, and synaptic reorganization, all of which contribute to heightened network excitability and seizure recurrence. In severe or prolonged cases, these changes can progress to status epilepticus or febrile seizures [22,23].

During early brain development, seizures exert profound effects on immature hippocampal circuits, interfering with synaptogenesis and disrupting the refinement of excitatory–inhibitory balance [20,24]. Given the hippocampus’s central role in both seizure generation and higher cognitive functions such as learning and memory, early-life seizures can produce long-lasting alterations in hippocampal circuitry. Recurrent or prolonged seizures during critical developmental windows impair cognitive processing and accelerate epileptogenic progression [20,22,24]. Specific developmental “critical periods” exist during which hippocampal neurons are particularly vulnerable to hyperexcitable activity; aberrant neurogenesis during these windows contributes to the establishment of pathological circuits and increased seizure susceptibility later in life [6,25].

Epileptic activity during hippocampal development disrupts normal patterns of neuronal maturation, synaptic connectivity, and circuit integration—processes essential for cognition, learning, and memory formation. Both in vivo and in vitro models demonstrate that seizure activity suppresses dendritic growth and branching of hippocampal pyramidal neurons, leading to reduced excitatory synaptic density and consequent cognitive deficits [26].

Early-life seizures perturb glutamatergic signaling and impair synaptic plasticity, particularly long-term potentiation (LTP) and long-term depression (LTD). These maladaptive receptor modifications compromise memory encoding and contribute to the pathogenesis of epilepsy later in life [24,26,27,28]. During critical developmental stages, recurrent seizures hinder the maturation of hippocampal circuitry, producing enduring deficits in spatial learning and memory [24].

Following severe brain injury, acute seizures frequently evolve into chronic epilepsy accompanied by cognitive impairment. Aberrant hippocampal neurogenesis is commonly observed under these conditions; however, the precise contribution of adult-born neurons to epileptogenesis and associated cognitive deficits remains incompletely understood [6]. Recurrent seizures produce marked deficits in learning and memory and significantly slow the growth of basilar dendrites, suggesting that persistent seizure activity in childhood epilepsy contributes to long-term impairments in cognition and hippocampal plasticity by suppressing dendritic development [29].

### 2.4. Modeling Epilepsy Using Hippocampal Organoid

Brain organoids are miniature, three-dimensional models of the human brain generated in vitro from pluripotent stem cells, including embryonic stem cells (ESCs) and induced pluripotent stem cells (iPSCs) [30]. They recapitulate key aspects of brain development, such as the formation of brain-like structures, differentiation into neurons and glial cells, and early neural network activity. These features make organoids valuable platforms for investigating both normal neurodevelopment and neurological disorders [31,32,33,34,35].

Among brain organoids, hippocampal organoids have attracted significant attention due to their ability to model region-specific processes and disease mechanisms, particularly in epilepsy. Development of hippocampal organoids typically involves stepwise protocols that include the selection of pluripotent cell sources, application of patterning factors, and validation of region-specific markers. In murine models, protocols using embryonic neural stem cells have shown that WNT3a signaling can promote hippocampal specification [36], with subsequent functional assays including calcium imaging and metabolic profiling to assess network activity [37].

Building on these animal studies, several groups have generated human hippocampal organoids from pluripotent stem cells [35,38,39,40,41,42]. Hippocampal induction is commonly achieved by activating WNT3a and SHH pathways [40], often followed by maturation with brain-derived neurotrophic factor (BDNF) [42]. Alternative approaches involve directing neural epithelium from anterior to dorsomedial forebrain regions using WNT and BMP signaling, recapitulating hippocampal development in vitro [41]. Several studies have attempted to generate hippocampus-like organoids, providing critical insights into the signaling mechanisms that drive stem cells toward a hippocampal identity [40,41,42]. Sakaguchi et al. first showed that WNT and BMP signaling induce a dorsomedial telencephalic (medial pallium) fate in human embryonic stem cells, resulting in 3D neural embryoid bodies containing hippocampal-like cells [41]. Pomeshchik et al. generated hippocampal spheroids from human iPSCs using the WNT agonist CHIR99021, which expressed general hippocampal markers (ZBTB20) and dentate gyrus-specific markers (PROX1) [42]. More recently, Wu et al. optimized the timing and combination of BMP, WNT, and SHH activation to enhance hippocampal neuron differentiation efficiency [40] (Supplementary Materials).

In human hippocampal organoids, the expression of characteristic markers such as Prox1, Zbtb20, Calbindin (CB), and Parvalbumin (PV) enables the identification of distinct hippocampal cell populations. Specifically, Prox1 marks dentate gyrus granule cells, Zbtb20 labels pyramidal neurons, CB indicates mature granule cells and interneurons, and PV identifies fast-spiking GABAergic interneurons. Neuronal maturation and synapse formation can be further assessed using NeuN, MAP2, and Synapsin-1, which reflect neuronal differentiation, dendritic development, and synaptogenesis, respectively. Collectively, these markers provide a robust framework for evaluating both the cellular composition and functional maturation of hippocampal organoids in vitro.

Human brain organoids have emerged as powerful experimental platforms that reproduce key aspects of human neurodevelopment and can be applied to the study of various neurological and neurodevelopmental disorders, including epilepsy [43]. Among these, hippocampal organoid models enable region-specific investigation of molecular and cellular mechanisms underlying epileptogenesis.

In studies using hippocampal organoids derived from patients carrying SCN8A variants, researchers observed pronounced disruptions in neural rhythms associated with learning and memory. These disturbances were attributed to a selective loss of inhibitory neurons within the hippocampus—often referred to as the brain’s ‘traffic cops’—which play a critical role in maintaining neural circuit stability. Such cellular alterations may underlie the cognitive and behavioral impairments commonly observed in epilepsy patients [30].

Recent studies have further expanded the application of human hippocampal organoids to investigate adult neurogenesis, network plasticity, and epilepsy-related neurodevelopmental abnormalities. Integration of multi-electrode array (MEA) technology enables real-time monitoring of neural firing patterns, network synchronization, and hyperexcitability, providing a functional platform to analyze aberrant circuitry characteristic of epilepsy [44,45]. Complementary techniques such as calcium imaging enable high-resolution visualization of intracellular calcium dynamics, revealing synchronized network bursts and hyperactive neuronal populations. Pharmacological testing with antiepileptic or excitatory/inhibitory modulators further allows evaluation of drug responsiveness, modulation of neuronal firing, and correction of abnormal network activity. Together, these approaches provide a multidimensional framework for assessing both the structural and functional maturation of hippocampal organoids and their application as in vitro models of epilepsy.

### 2.5. Genes and Neurotransmitter Markers in the Hippocampus Related to Epilepsy

Hippocampal organoid models have been used to analyze epilepsy-associated genes and neurotransmitter markers to investigate the molecular mechanisms underlying hippocampus-related epilepsy. Among genetic factors, SCN8A, encoding the voltage-gated sodium channel Nav1.6, is critical for neuronal excitability. Gain-of-function mutations in SCN8A cause developmental and epileptic encephalopathy type 13 (DEE13), characterized by early-onset seizures, intellectual disability, and autism spectrum disorder [46]. Patient-derived hippocampal organoids carrying SCN8A mutations exhibit disrupted neural rhythmicity associated with learning and memory deficits. These disturbances are primarily attributed to a selective loss of inhibitory neurons within the hippocampus, which are critical for maintaining neural circuit stability, providing a mechanistic explanation for cognitive impairments beyond seizure activity. Further studies indicate that SCN8A mutations induce network hyperexcitability in cortical models while causing region-specific dysregulation in hippocampal networks, underscoring the importance of region-dependent disease mechanisms in epilepsy [46].

Additionally, SCN1A and SCN2A—encoding other voltage-gated sodium channel subunits—were included in the analysis due to their established roles in modulating inhibitory-excitatory balance in hippocampal circuits [47,48]. Previous studies using animal models (mouse and Drosophila) and patient-derived iPSC organoids and assembloids carrying SCN1A, SCN2A, or SCN8A mutations effectively recapitulate the cellular, molecular, and electrophysiological mechanisms underlying developmental epilepsies [49,50,51,52]. Specifically, patient-derived organoid studies have revealed variant-specific alterations in neuronal excitability, chromatin remodeling, synaptic development, and differential pharmacological responses, for example, to phenytoin. Furthermore, assembloid models demonstrate that microglia selectively respond to hyperexcitable mutant neurons, highlighting the importance of cell-type-specific interactions in disease pathophysiology. Collectively, these models not only provide mechanistic insights into sodium channel–related epilepsies but also offer a valuable platform for evaluating personalized therapeutic strategies, underscoring their translational potential in precision medicine.

To further characterize neuronal subtypes within hippocampal organoids, a range of neurotransmitter markers have been examined. Excitatory (glutamatergic) neurons were identified using vesicular glutamate transporters (VGLUT1 and VGLUT2) and NMDAR1, which are critical for synaptic plasticity and memory-related signaling. Inhibitory (GABAergic) interneurons were labeled using parvalbumin (PV), calbindin (CB), calretinin (CR), neuropeptide Y (NPY), somatostatin (SOM), and vasoactive intestinal peptide (VIP) [53,54]. Choline acetyltransferase (ChAT) was used to identify cholinergic neurons predominantly located in the CA4 and dentate gyrus regions [55].

Additional analysis of epilepsy-related neurotransmission included GABAA receptor subunits (GABRA1 and GABRG2), AMPA receptor auxiliary proteins (GRIA1, GRIN2A, SHISA9, and CACNG3), and voltage-gated calcium channel subunits (CACNA2D2 and α2δ-2) [56,57,58,59]. Moreover, ARX, a transcription factor critical for interneuron development and GABAergic neurotransmission, was examined to assess its contribution to interneuron specification and network regulation [60,61].

### 2.6. Therapeutic Strategies and Future Directions for Epilepsy in the Developing Hippocampus

Recent advances in the treatment of developmental hippocampal epilepsy encompass a broad spectrum of strategies targeting both seizure control and underlying pathophysiological mechanisms. Conventional approaches include pharmacological therapies with antiepileptic drugs (AEDs) and surgical interventions such as laser interstitial thermal therapy (LITT) [62,63]. In addition, neuromodulation techniques, including vagus nerve stimulation (VNS) and deep-brain stimulation (DBS), have emerged as promising modalities for patients with drug-resistant epilepsy involving hippocampal circuits [61].

Emerging and experimental therapies increasingly leverage brain organoid models to elucidate disease mechanisms and develop targeted interventions. Approaches under investigation include gene therapy, neurosteroid-based modulation, and epigenetic modification strategies aiming to restore normal neuronal excitability and circuit function [64]. Precision medicine initiatives seek to tailor treatments to individual genetic and neurochemical profiles, thereby enhancing efficacy and specificity [63,64].

Hippocampal organoid models have proven particularly valuable for studying developmental epileptic encephalopathy associated with specific gene variants. For example, studies from UCLA demonstrated that patient-derived organoids carrying disease-causing variants exhibit disrupted neural activity and connectivity, resulting in seizure-like discharges and impaired network function [46]. These models enable a detailed investigation of how specific genetic alterations perturb hippocampal circuits and contribute to cognitive dysfunction [44,65]. Electrophysiological analyses further reveal region-specific seizure phenotypes and neuronal hyperexcitability linked to mutations or developmental abnormalities, offering critical insights for drug discovery and the development of personalized therapies [66].

Looking forward, insights gained from hippocampal organoid studies may be translated into early intervention strategies in pediatric populations. By modeling brain development at different stages, organoid-based research has the potential to prevent or mitigate neurological disorders across the lifespan, ultimately improving quality of life. This framework supports the advancement of personalized medicine and the development of targeted therapeutic strategies for neurodevelopmental conditions. Moreover, combinatorial approaches integrating pharmacological, surgical, and neuromodulatory interventions hold promise for enhancing outcomes in refractory cases.

## 3. Discussion

The hippocampus plays a critical role in various cognitive functions, including learning and memory, serving as a central structure for the integration and consolidation of information within the brain. Proper development of the hippocampus is essential, as disturbances during this process can have profound and lasting effects on cognitive and behavioral outcomes. In particular, damage to the immature hippocampus caused by epileptic activity can lead to severe and often irreversible impairments, underscoring the importance of identifying strategies to prevent or mitigate such injury.

Beyond its well-established roles in spatial and declarative memory, recent evidence suggests that the hippocampus also contributes to social cognition by constructing “social maps” that encode relationships and social interactions [7,9,67,68]. These broader functions position the hippocampus as a multidimensional integrative hub, involved not only in memory processing but also in the representation of complex cognitive and social contexts.

The prolonged and region-specific maturation of hippocampal subfields reflects the intricate complexity of the neural circuitry that underlies cognitive and mnemonic processes. Extended neurogenesis in the DG and late myelination in regions such as CA1 and the subiculum indicate sustained plasticity that supports learning and memory across development. The asynchronous maturation of these subregions may explain the gradual refinement of spatial navigation, associative learning, and episodic memory observed throughout childhood and adolescence. Collectively, these developmental dynamics highlight the hippocampus as a continuously evolving structure whose maturation parallels the emergence of higher cognitive functions.

The extended maturation of hippocampal subfields, particularly CA1 and DG, underscores the persistence of neural plasticity and structural remodeling beyond early childhood. This prolonged development may account for the hippocampus’s lifelong adaptability in learning and memory. Conversely, age-related reductions in hippocampal volume—especially in CA1 and CA2- have been associated with cognitive decline in later life, suggesting that the same plasticity that supports learning during development may also confer vulnerability to degeneration in aging.

Importantly, the hippocampus plays a pivotal role in both the initiation and progression of seizures and is particularly vulnerable to seizure-induced disruption during development. Such disturbances may underlie the long-lasting cognitive deficits frequently observed in individuals with early-life epilepsy [6,20,22,24,25,69]. The suppression of dendritic growth and the disorganization of synaptic connections suggest that recurrent seizures impair hippocampal network formation and plasticity during critical developmental periods.

Moreover, aberrant hippocampal neurogenesis observed following epileptic insults appears to be a key factor contributing to chronic seizure susceptibility and associated memory impairments. Targeting this maladaptive neurogenesis, while promoting normal synaptic and dendritic development, may represent a promising therapeutic strategy to reduce seizure recurrence and restore cognitive function after epileptic injury.

The present findings indicate that hippocampal dysfunction in epilepsy involves alterations in specific interneuron populations and glutamate receptor expression, which together disturb excitatory–inhibitory balance. In particular, GABA*_A_* receptor subunits, including GABRA1 (α1) and GABRG2 (γ2), represent critical molecular determinants of inhibitory neurotransmission, which is essential for the regulation of neuronal excitability and seizure control [70,71]

Moreover, ARX mutations contribute to defective interneuron differentiation and impaired GABAergic signaling within the hippocampus, strongly correlating with epilepsy phenotypes [60,61]. These observations suggest that both genetic factors and neurotransmitter-level disruptions converge on impaired inhibitory circuitry as a central mechanism underlying hippocampal epileptogenesis.

Despite advances achieved through hippocampal organoid studies, current models still exhibit only partial induction of hippocampal identity. Although general hippocampal markers (e.g., ZBTB20) and dentate gyrus markers (e.g., PROX1 andLEF1) are expressed, the molecular features and structural organization characteristic of distinct hippocampal subfields—such as CA1, CA2, and CA3—remain largely absent. This lack of regional specification and the incomplete recapitulation of the temporal dynamics of human hippocampal development represent major limitations in faithfully modeling hippocampal function in vitro.

Nevertheless, insights gained from research on hippocampal organoids are expected to deepen our understanding of epilepsy mechanisms and to promote the development of more precise and effective therapeutic strategies. By integrating electrophysiological, molecular, and genetic data, organoid-based systems may help identify novel therapeutic targets and facilitate personalized medicine approaches tailored to individual patient profiles. In the future, these findings may be translated into clinical applications for young children at various stages of brain development. Early intervention based on organoid-derived insights could help prevent or mitigate a broad spectrum of neurodevelopmental and neurological disorders throughout life, thereby improving long-term quality of life. Furthermore, combining pharmacological, surgical, and neuromodulation-based strategies with organoid-guided precision medicine holds considerable promise for advancing individualized treatment paradigms in intractable epilepsy and related neurodevelopmental conditions.

### Limitations

Although hippocampal organoids have emerged as promising in vitro systems for modeling epilepsy and investigating disease-related mechanisms, several critical limitations remain. First, current organoids have not yet achieved full structural and functional fidelity to the human hippocampus. Most studies are confined to molecular-level analyses and fail to recapitulate the complete cytoarchitecture, partly because hippocampal development continues postnatally, making it challenging to generate fully mature hippocampal models in vitro. Second, due to the absence of subpallial regions, essential neuronal populations—such as GABAergic interneurons that normally originate in the subpallium and migrate into the hippocampus—are largely lacking, further restricting the organoids’ capacity to model mature hippocampal function and epileptogenesis.

Additional technical limitations include batch-to-batch variability, which reduces experimental reproducibility, and the lack of vascularization and myelination, preventing organoids from fully recapitulating the in vivo brain environment. Moreover, developmental immaturity constrains their ability to faithfully replicate the structural and functional characteristics of the adult human hippocampus. Finally, ethical considerations arise from the use of human-derived cells, particularly as brain organoids may partially recapitulate neural activity and cognitive-like functions, necessitating careful experimental design, monitoring, and oversight in both research and potential translational applications.

Collectively, these limitations underscore the need for continued refinement of hippocampal organoid models, including improved regional specification, enhanced maturation, and incorporation of supporting systems such as vascular networks, to increase their utility as translational models for epilepsy and other neurodevelopmental disorders.

## 4. Conclusions and Future Directions

Epilepsy-induced damage to the developing hippocampus can compromise its core functions, including short- and long-term memory, spatial navigation, and overall cognitive abilities, highlighting the critical need for strategies to prevent or mitigate such impairments. Hippocampal organoids offer a promising in vitro platform for investigating mechanisms underlying epilepsy and the complex interactions among molecular and cellular factors, with the ultimate aim of identifying novel therapeutic approaches.

Despite recent advances, research in this field remains in its early stages. Currently, no standardized and reproducible protocol exists for generating structurally and functionally mature hippocampal organoids, and this limitation hinders their application in studying hippocampus-related disorders, including epilepsy.

Future research should focus on optimizing differentiation protocols and integrating advanced technologies such as single-cell transcriptomics, electrophysiological mapping, and assembloid systems to enhance organoid fidelity and functional maturation. Furthermore, identifying epilepsy-associated genetic and molecular factors and exploring strategies to modulate or rescue their effects will be essential. By addressing these challenges, hippocampal organoids have the potential to serve as a powerful experimental platform for developing fundamental therapeutic strategies for epilepsy and for preserving cognitive function.

## Data Availability

No new data were created or analyzed in this study. Data sharing is not applicable to this article.

## Supplementary Materials

The following supporting information can be downloaded at: https://www.mdpi.com/article/10.3390/brainsci15111231/s1, Table S1: Overview of Experimental Approaches for Hippocampal Organoid Generation.

## Abbreviations

The following abbreviations are used in this manuscript

TLE	Temporal Lobe Epilepsy
DG	Dentate Gyrus
CA	Cornu Ammonis
HFOs	High-Frequency Oscillations
hPSC	Human Pluripotent Stem Cell
Wnt3a	Wingless -Type MMTV Integration Site Family, Member 3A
SHH	Sonic Hedgehog
ZBTB20	Zinc Finger and BTB Domain Containing 20
PROX1	Prospero Homeobox 1
CB	Calbindin
CR	Calretinin
NPY	Neuropeptide R
SOM	Somatostatin
VIP	Vasoactive Intestinal Peptide
iPSCs	Induced Pluripotent Stem Cells
SCN8A	Sodium Voltage-Gated Channel Alpha Subunit 8
LTP/LTD	Long-Term Potentiation/Long-Term Depression
EDD-13	Epileptic Encephalopathy Type 13
NeuN	Neuronal Nuclei
MAP2	Microtubule-Associated Protein 2
VGLUT	Vesicular Glutamate Transporters
NMDA	N-Methyl- D-Aspartate
GABA	Gamma-Aminobutyric Acid
ARX	Aristaless-Related Homeobox (gene regulating brain development and interneuron differentiation)
PV	Parvalbumin
GRIA1	Glutamate Ionotropic Receptor AMPA Type Subunit 1
GRIN2A	Glutamate Ionotropic Receptor NMDA Type Subunit 2A
SHISA9	Shisa Family Member X
CACNG	Calcium Voltage Gated Channel Auxiliary Subunit (neuronal AMPA receptor modulation)
LITT	Laser Interstitial Thermal Therapy
ChAT	Choline Acetyltransferase
VNS	Vagus Nerve Stimulation
DBS	Deep Brain Stimulation
