# Supplementary material for: Hippocampal Development and Epilepsy: Insights from Organoid Models
Source: Brain Sci. 2025 Nov 16;15(11):1231. doi: 10.3390/brainsci15111231 (PMC12651821; doi:10.3390/brainsci15111231)
Supplement: Supplementary file 1 [file brainsci-15-01231-s001.zip › brainsci-3948000-supplementary.pdf]

## Table S1. Overview of Experimental Approaches for Hippocampal Organoid Generation

| | Cell sources | | Inducing factors | Culture duration | Validation markers & electrophysiological methods | Main finding and limitation |
|---|---|---|---|---|---|---|
| Sakaguchi et al. (*Nature Commun*. 2015)[41] | hESCs | Neural induction | Wnt3, SB431542(TGF-β inhibitor) | 0 ~ 18 days | PROX1, ZBTB20, KA1 (Dentate gyrus granule) | **Main findings**: Simultaneous activation of WNT and BMP signaling → self-organization of dorsomedial telencephalic tissue from hESCs → generation of hippocampal neuron (PROX1+, ZBTB20+, KA1+) and the establishment of functional synaptic activity. |
| | | Dorsomedial induction | CHIR99021 (3 μM) + BMP4 (0.5 nM) + 10% FBS | 18 ~21 days | Whole-cell patch-clamp recording/ Calcium imaging/Synaptic connectivity assays | **Limitation:** Structural immaturity, incomplete circuit functionality, limited cellular diversity, difficulty in long-term culture, and lack of in vivo validation. |
| | | Maintenance/ maturation | Neurobasal medium supplemented with B27 | 21~100+ days | | |

| | | | | | | |
|---|---|---|---|---|---|---|
| Sarkar A. et al., (*Cell Stem Cell* 2018)[39] | hESCs, hiPSCs | Stepwise hippocampal differentiation | dual-SMAD inhibition followed by WNT and SHH pathway modulation:<br>•BMP/TGF-β inhibition (Noggin, SB431542)<br>•WNT3A and FGF8 to induce medial pallium identity<br>•Subsequent WNT activation promotes CA3 pyramidal neuron fate. | Approximately 4–5 weeks in vitro (WIV**)** | CA3 markers**: GRIK4 (KA1), SCGN**(Secretagogin), **ELAVL2.**<br>Neuronal markers: **MAP2, NeuN.**<br>Forebrain markers: **FOXG1, OTX1/2.**<br><br>Validation by immunostaining, qRT-PCR, and RNA profiling | **Main finding:**<br>functional human CA3 pyramidal neurons, DG–CA3 synaptic connectivity in vitro, reduced neuronal activity in schizophrenia |
| | | | | | Whole-cell patch-clamp recording, Spontaneous and evoked EPSCs, Monosynaptic rabies virus tracing | **Limitations:**<br>CA3 structural and functional immaturity, Incomplete long-term maturation, Variability among hPSC lines |
| Pomeshchik et al., (*Stem Cell Reports*,2020)[42] | hiPSCs | Neural induction | SB431542 (TGF-β inhibitor) and LDN193189 (BMP inhibitor) | Approximately 70–90 days | Neural progenitor markers: **SOX2, PAX6.**<br>Hippocampal regional markers: **PROX1, ZBTB20, FOXG1, LHX2.**<br>Neuronal maturation markers**: MAP2, NeuN, SYN1** (Synapsin-1).<br>Astrocytic marker: **GFAP** | **Main Findings:**<br>3D hippocampal spheroids, AD patient-specific phenotypes, a disease-modeling and drug-screening platform for Alzheimer’s disease |

| | | | | | | |
|---|---|---|---|---|---|---|
| | | Hippocampal patterning | BDNF and GDNF to promote hippocampal neuron maturation.<br>FGF2, CHIR99021 (GSK3β inhibitor), and BMP7 | | Patch-clamp electrophysiology, Calcium imaging, Amyloid-β and Tau assays | **Limitations:**<br>Incomplete structural organization, Limited cellular diversity, Variability, Lack of optimization of long-term functional stability |
| Ciarpella et al., (iScience 2021)[36] | NSCs | Growth phase | EGF (20 ng/mL), FGF2 (20 ng/mL) | Approximately 50–60 days | Neuronal and regional markers**:** **MAP2, NeuN, TBR1, PROX1, ZBTB20, DCX.**<br>Synaptic markers: **Synapsin-1, PSD95**.<br>Proliferation marker**:** **Ki67** | **Main findings:**<br>Established a murine cerebral organoid model (hippocampal-like subregions)<br>functional neuronal networks, evidenced by spontaneous calcium oscillations, synaptic activity, and action potential firing, hippocampal-specific markers (PROX1, ZBTB20), intrinsic patterning mechanisms |
| | | Differentiation phase | Neurobasal + B27 supplement (without EGF/FGF2) | | | **Limitations:**<br>Incomplete laminar and cytoarchitectural organization,<br>Limited maturation,<br>Absence of vascularization and external inputs,<br>Variability in organoid size and regional identity |

| | | | | | | |
|---|---|---|---|---|---|---|
| Ciarpella F. et al., (*STAR Protocols* 2023)[37] | NSCs | Expansion | bFGF/EGF → NSC proliferation | 0~4 days | Pan-hippocampal marker: **ZBTB20**. CA3 marker: **KA1 (GRIK4)**. CA2 marker: **FZD9**. General neuronal marker: **MAP2**. | **Main findings**: rapid (~1 month), reproducible protocol, hippocampal region specification using WNT3a, hippocampal marker expression (ZBTB20, KA1) at ~32 days |
| | | Induction | decreasing bFGF/EGF addition of Wnt3a | 5~14 days | Calcium imaging, Metabolic profiling, Immunofluorescence analysis | **Limitations:** not fully validate long-term functional maturation, translational limitation. Structural and cell-type diversity may be limited |
| | | Differentiation | | 15~32 days | | |
| Wu Y. et al., (*Nature Communications* 2024)[40] | hiPSCs → hHOs | Organoid differentiation and maturation | Wnt3a/ SHH activator | Approximately 90 days | Progenitors; **HOPX, PAX6**. Neurons; **PROX1, ZBTB20** (dentate gyrus granule neurons). Neuronal maturation; **MAP2, NeuN,** Glial**;** **GFAP** (astrocytes) | **Main findings:** 3D hippocampal organoid model (developing human hippocampus), stretchable, conformal liquid-metal mesh neuro-interface, functional network activity and region-specific hippocampal identity |
| | | Characterization | | Day 30, 60, 90 | | |
| | | scRNA-seq | | Day 81 | mMPC; spontaneous spikes (50–150 μV), bursting activity, and network oscillations in 3D hHOs. | **Limitation**: structural and circuit immaturity, Mechanical pressure or diffusion limitation, Limited long-term metabolic support, Incomplete in vivo validation |

**hiPSCs**; Human induced pluripotent stem cells, **hHOs**; human hippocampal organoids, **hESC**: human embryonic stem cells, **NSCs**; Mouse embryonic neural stem

cells, **mMPC**; 3D liquid-metal mesh multi-electrode array, **scRNA-seq**; single-cell RNA sequencing,
**bFGF**; *basic Fibroblast Growth Factor* (also known as FGF2), **EGF**; Epidermal Growth Factor
**HOPX;** HOP Homeobox**, PAX6**; *Paired Box 6*, **GFAP**; Glial Fibrillary Acidic Protein, **PROX1**; Prospero Homeobox 1, **ZBTB20**; Zinc Finger and BTB Domain Containing 20**, MAP2**; Microtubule-Associated Protein 2, **NeuN**; *Neuronal Nuclei*
**Wnt3a**; Wingless-Type MMTV Integration Site Family, Member 3A, **SHH**; Sonic Hedgehog, **BDNF;** Brain-Derived Neurotrophic Factor, **GDNF;** Glial cell line-Derived Neurotrophic Factor, **BMP7;** Bone Morphogenetic Protein 7, **TGF-β;** Transforming Growth Factor-Beta
